# Elranatamab in relapsed or refractory multiple myeloma: the MagnetisMM-1 phase 1 trial

**DOI:** 10.1038/s41591-023-02589-w

**Published:** 2023-10-02

**Authors:** Nizar J. Bahlis, Caitlin L. Costello, Noopur S. Raje, Moshe Y. Levy, Bhagirathbhai Dholaria, Melhem Solh, Michael H. Tomasson, Michael A. Damore, Sibo Jiang, Cynthia Basu, Athanasia Skoura, Edward M. Chan, Suzanne Trudel, Andrzej Jakubowiak, Cristina Gasparetto, Michael P. Chu, Andrew Dalovisio, Michael Sebag, Alexander M. Lesokhin

**Affiliations:** 1 Arnie Charbonneau Cancer Institute, University of Calgary, Calgary, AB Canada; 2 Moores Cancer Center, University of California, San Diego, La Jolla, CA USA; 3 Massachusetts General Hospital Cancer Center, Harvard Medical School, Boston, MA USA; 4 Department of Medical Oncology, Baylor Scott and White Health, Dallas, TX USA; 5 Vanderbilt-Ingram Cancer Center, Nashville, TN USA; 6 Blood and Marrow Transplant Group of Georgia, Northside Hospital, Atlanta, GA USA; 7 Holden Comprehensive Cancer Center, University of Iowa, Iowa City, IA USA; 8 Oncology Research and Development, Pfizer Inc., San Diego, CA USA; 9 Early Clinical Development, Pfizer Inc., San Diego, CA USA; 10 Early Clinical Development, Pfizer Inc., Collegeville, PA USA; 11 Oncology Research and Development, Pfizer Inc., South San Francisco, CA USA; 12 Princess Margaret Cancer Centre, University Health Network, Toronto, ON Canada; 13 Department of Medicine, University of Chicago Medical Center, Chicago, IL USA; 14 Department of Medicine, Duke University Cancer Institute, Durham, NC USA; 15 Cross Cancer Institute, Edmonton, AB Canada; 16 Department of Hematology and Oncology, Ochsner Health, New Orleans, LA USA; 17 Cedars Cancer Center, McGill University Health Center, Montreal, QC Canada; 18 Division of Hematology and Oncology, Memorial Sloan Kettering Cancer Center/Weill Cornell Medical College, New York, NY USA

**Keywords:** Myeloma, Myeloma

## Abstract

Multiple myeloma (MM) is a plasma cell malignancy expressing B cell maturation antigen (BCMA). Elranatamab, a bispecific antibody, engages BCMA on MM and CD3 on T cells. The MagnetisMM-1 trial evaluated its safety, pharmacokinetics and efficacy. Primary endpoints, including the incidence of dose-limiting toxicities as well as objective response rate (ORR) and duration of response (DOR), were met. Secondary efficacy endpoints included progression-free survival (PFS) and overall survival (OS). Eighty-eight patients with relapsed or refractory MM received elranatamab monotherapy, and 55 patients received elranatamab at efficacious doses. Patients had received a median of five prior regimens; 90.9% were triple-class refractory, 29.1% had high cytogenetic risk and 23.6% received prior BCMA-directed therapy. No dose-limiting toxicities were observed during dose escalation. Adverse events included cytopenias and cytokine release syndrome. Exposure was dose proportional. With a median follow-up of 12.0 months, the ORR was 63.6% and 38.2% of patients achieving complete response or better. For responders, the median DOR was 17.1 months. All 13 patients evaluable for minimal residual disease achieved negativity. Even after prior BCMA-directed therapy, 53.8% achieved response. For all 55 patients, median PFS was 11.8 months, and median OS was 21.2 months. Elranatamab achieved durable responses, manageable safety and promising survival for patients with MM. ClinicalTrials.gov Identifier: NCT03269136.

## Abstract (web summary)

In the first-in-human trial of elranatamab, patients with refractory or relapsed multiple myeloma who received the bispecific antibody against BCMA and CD3 experienced no dose-limiting toxicities during dose escalation, and the agent showed promising clinical efficacy.

## Main

Multiple myeloma (MM) is an incurable plasma cell malignancy. Clinical outcomes remain poor for patients with relapsed or refractory multiple myeloma (RRMM) after therapy with at least one proteasome inhibitor, one immunomodulatory drug and one CD38-directed antibody^1^. There remains a substantial need to develop novel therapeutic approaches to improve outcomes for patients. B cell maturation antigen (BCMA), a member of the tumor necrosis factor receptor family, represents a promising target due to its downstream signaling through survival pathways and its ubiquitous expression on the surface of myeloma cells^2,3^. In addition, soluble BCMA is elevated in the sera of patients with MM and correlates with disease burden and survival^4^.

Novel BCMA-directed therapies include antibody–drug conjugates (ADCs), chimeric antigen receptor T cell (CAR-T) therapies and T-cell-engaging bispecific antibodies. In the United States, three BCMA-targeted therapies are currently approved to treat patients with RRMM. Idecabtagene vicleucel (ide-cel) and ciltacabtagene autoleucel (cilta-cel), both CAR-T-cell therapies, showed an objective response rate (ORR) of 67.1% (94/140) and 83.2% (94/113), respectively, in patients who underwent leukapheresis^5–7^. Teclistamab, a T-cell-engaging bispecific antibody, demonstrated an ORR of 63.0% (104/165) (refs. ^8,9^).

Elranatamab (PF-06863135) is a humanized bispecific IgG2 antibody targeting BCMA on myeloma cells and CD3 on T cells. Elranatamab activates and directs T cells to induce a selective cytotoxic T cell response against myeloma cells^10^. In preclinical models, elranatamab induced dose-dependent death of myeloma cell lines and primary patient cells as well as tumor regression in orthotopic myeloma xenograft models^10^. Here we present results from the ongoing first-in-human phase 1 study (MagnetisMM-1) evaluating the safety, pharmacokinetics, pharmacodynamics and efficacy of elranatamab for patients with RRMM. With the exception of safety, this report focuses primarily on evaluating outcomes for 55 patients with RRMM who received single-agent elranatamab subcutaneously at efficacious doses ≥215 µg kg^−1^.

## Results

### Trial design and patients

**Fig. 1:**
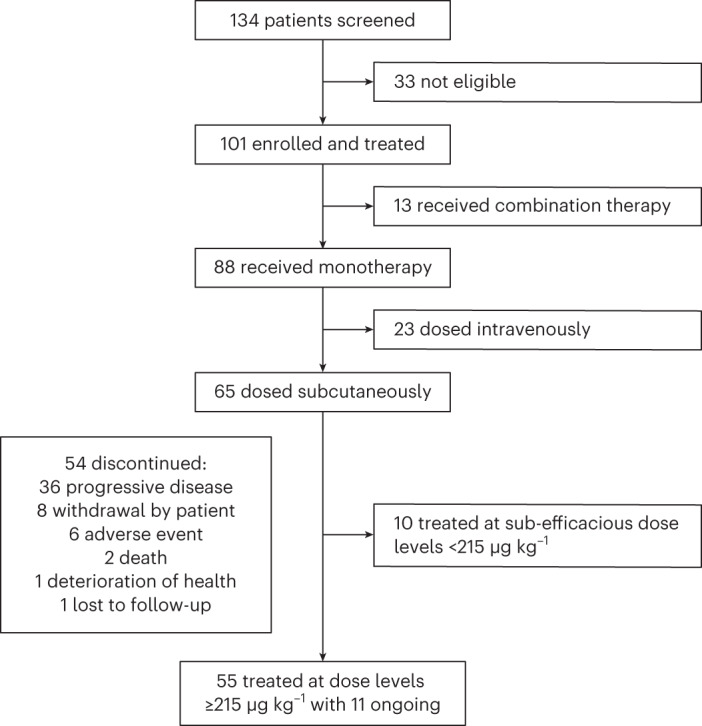
CONSORT diagram of MagnetisMM-1.

Between 29 November 2017 and 8 April 2021, 134 patients were screened, and 101 were enrolled and received at least one dose of elranatamab (Fig. 1). Of the 101 patients, 88 received elranatamab monotherapy either intravenously (*n* = 23) or subcutaneously (*n* = 65). Of the 65 patients who received elranatamab subcutaneously and with a data cutoff of 30 September 2022, 11 patients were ongoing and 54 had discontinued due to progressive disease (*n* = 36), withdrawal by patient (*n* = 8), adverse event (*n* = 6), death (*n* = 2), deterioration of health (*n* = 1) or lost to follow-up (*n* = 1). For subcutaneous monotherapy, 10 patients received elranatamab at sub-efficacious dose levels (80 μg kg^−1^ or 130 μg kg^−1^) not associated with International Myeloma Working Group (IMWG)-confirmed responses of partial response (PR) or better, and 55 patients received elranatamab at efficacious dose levels ≥215 μg kg^−1^. For these 55 patients, median age was 64 years (range, 42‒80) with 29 (52.7%) male and 26 (47.3%) female patients (Table 1). Notably, 17 (30.9%) patients had extramedullary disease, and 16 (29.1%) patients had a high cytogenetic risk at study entry based on local assessment and defined by the presence of detectable t(4;14), t(14;16) or del(17p) abnormalities. These patients had a median of five prior anti-myeloma therapies (range, 2‒14), and 38 (69.1%) patients had received prior stem cell transplants. A total of 54 (98.2%) patients had disease that was triple-class exposed, and 50 (90.9%) patients had disease that was triple-class refractory; 43 (78.2%) patients had disease that was penta-drug exposed, and 32 (58.2%) patients had disease that was penta-drug refractory. A total of 13 (23.6%) patients were exposed to prior BCMA-directed therapy, including ADCs in four (7.3%) patients, CAR-T therapy in five (9.1%) patients and both in four (7.3%) patients.

**Table 1:** Baseline characteristics and prior treatments

	Elranatamab SC monotherapy (*n* = 55)
Median age, years	64.0 (42–80)
Sex	
Female	26 (47.3)
Male	29 (52.7)
Race	
White	37 (67.3)
Black/African American	11 (20.0)
Asian	4 (7.3)
Not reported	3 (5.5)
ECOG PS	
0–1	50 (90.9)
≥2	5 (9.1)
R-ISS stage at initial diagnosis	
Stage I	14 (25.5)
Stage II	20 (36.4)
Stage III	11 (20.0)
Not reported	10 (18.2)
Cytogenetic risk	
High^a^	16 (29.1)
Standard	35 (63.6)
Unknown	4 (7.3)
Extramedullary disease	17 (30.9)
Median number of prior anti-myeloma therapies	5.0 (2–14)
Triple-class refractory^b^	50 (90.9)
Refractory to last line of therapy	49 (89.1)
Prior PIs	55 (100.0)
Bortezomib	52 (94.5)
Carfilzomib	47 (85.5)
Ixazomib	18 (32.7)
Prior ImiDs	55 (100.0)
Lenalidomide	54 (98.2)
Pomalidomide	52 (94.5)
Thalidomide	9 (16.4)
CC-92480	2 (3.6)
Iberdomide	1 (1.8)
Prior anti-CD38 therapy	54 (98.2)
Daratumumab	52 (94.5)
Isatuximab	4 (7.3)
Other^c^	1 (1.8)
Prior BCMA-targeted therapy	13 (23.6)
Anti-BCMA ADC	4 (7.3)
CAR-T	5 (9.1)
Both anti-BCMA ADC and CAR-T	4 (7.3)

Values are median (range) or *n* (%). Data cutoff was 30 September 2022. Patients may have received more than one treatment within a given therapy class.

^a^Definition of high cytogenetic risk includes t(4;14), t(14;16) and del(17p).

^b^Triple-class refractory disease is refractory to at least one PI, one ImiD and one anti-CD38 therapy.

^c^One patient treated at 360 μg kg^−1^ received prior anti-myeloma therapy with a CD38×CD3 bispecific molecule.

ImiD, immunomodulatory drug; PI, proteasome inhibitor; R-ISS, Revised International Staging System; SC, subcutaneous.

### Safety endpoints

Elranatamab monotherapy demonstrated a manageable safety profile (*n* = 88) (Extended Data Table 1). No dose-limiting toxicities (DLTs) were observed during dose escalation (part 1), and the maximum tolerated dose of elranatamab was not reached. Among the 55 patients treated at doses associated with clinical efficacy, the most common treatment-emergent adverse events (TEAEs) regardless of causality (and irrespective of whether priming and premedication were implemented) included cytopenias, cytokine release syndrome (CRS) and injection site reaction (*n* = 55) (Table 2). Hematologic TEAEs were common and included neutropenia in 41 (74.5%), anemia in 37 (67.3%), lymphopenia in 29 (52.7%) and thrombocytopenia in 28 (50.9%) patients. The most common (≥50%) non-hematologic TEAEs were CRS in 48 (87.3%) patients and injection site reaction in 31 (56.4%) patients. CRS was limited to grades 1 and 2, with median time to onset of 1.0 d (range, 1.0‒3.0) and a median duration of 3.0 d (range, 1.0‒10.0); no grade ≥3 events were observed. For patients who received a priming dose of elranatamab, CRS occurred primarily with the priming dose; only two (6.1%) patients experienced grade 1 CRS after receiving the recommended phase 2 dose (RP2D). Among the 15 patients who received both a priming dose and dexamethasone-based premedication in part 2A, the overall incidence of CRS was reduced to 10 (66.7%) patients and limited to grade 1 in five (33.3%) patients and grade 2 in five (33.3%) patients; no grade ≥3 events were observed (Extended Data Table 2).

**Table 2:** TEASs (all causality)

Adverse event	Elranatamab SC monotherapy (*n* = 55)				
	Grade 1	Grade 2	Grade 3	Grade 4	Total
Hematologic					
Neutropenia	0	2 (3.6)	14 (25.5)	25 (45.5)	41 (74.5)
Anemia	1 (1.8)	8 (14.5)	28 (50.9)	0	37 (67.3)
Lymphopenia	0	1 (1.8)	3 (5.5)	25 (45.5)	29 (52.7)
Thrombocytopenia	6 (10.9)	6 (10.9)	6 (10.9)	10 (18.2)	28 (50.9)
Non-hematologic					
CRS^a^	28 (50.9)	20 (36.4)	0	0	48 (87.3)
Injection site reaction	27 (49.1)	4 (7.3)	0	0	31 (56.4)
Fatigue	7 (12.7)	12 (21.8)	4 (7.3)	0	23 (41.8)
Diarrhea	12 (21.8)	8 (14.5)	2 (3.6)	0	22 (40.0)
Dry skin	18 (32.7)	2 (3.6)	0	0	20 (36.4)
Hypophosphatemia	0	6 (10.9)	13 (23.6)	1 (1.8)	20 (36.4)
Decreased appetite	11 (20.0)	7 (12.7)	1 (1.8)	0	19 (34.5)
Nausea	6 (10.9)	10 (18.2)	3 (5.5)	0	19 (34.5)

Values are *n* (%). Data cutoff was 30 September 2022. Any grade TEAEs reported in more than 33.3% of patients. Grading of TEAEs was based on NCI CTCAE version 4.03, except for CRS. Grading of CRS was based on Lee et al.^13^.

^a^Twenty patients received no priming or premedication; 20 patients received priming only; and 15 patients received priming plus premedication. In the group that received priming plus premedication, the overall incidence of CRS was 67% and limited to grade 1 (33%) and grade 2 (33%), with seven (47%) patients receiving tocilizumab.

SC, subcutaneous.

In addition to the most common TEAEs, other adverse events were reported. Immune effector cell-associated neurotoxicity syndrome (ICANS) was limited to grade 1 in four (7.3%) patients and grade 2 in five (9.1%) patients; no grade ≥3 events were observed. Among the 15 patients who received both a priming dose and dexamethasone-based premedication in part 2A, ICANS was limited to grade 1 in one (6.7%) patient and grade 2 in one (6.7%) patient; no grade ≥3 events were observed (Extended Data Table 3). Infections of any etiology (including bacterial, viral and fungal) or grade were reported in 41 (74.5%) patients, with grade 3 events in 12 (21.8%) and grade 4 events in three (5.5%) patients. Among the 55 patients, 18 (32.7%) received intravenous immunoglobulin. Opportunistic infections occurred in five (9.1%) patients and included pneumocystis jirovecii pneumonia (*n* = 2, both grade 2), adenovirus infection (*n* = 1, grade 5), cytomegalovirus infection (*n* = 1, grade 2), cytomegalovirus infection reactivation (*n* = 1, grade 1) and pneumonia cytomegaloviral (*n* = 1, grade 1). There were eight (14.5%) deaths considered unrelated to study treatment, including three (5.5%) due to disease progression, two (3.6%) due to coronavirus disease 2019 (COVID-19) infection and one each due to plasma cell myeloma, sudden death and septic shock; one death due to adenovirus infection was assessed as related to study treatment. A total of eight (14.5%) patients discontinued treatment due to an adverse event, including one patient with pre-existing peripheral neuropathy who developed muscular weakness assessed as related to study treatment.

### Pharmacokinetics and pharmacodynamics

**Fig. 2:**
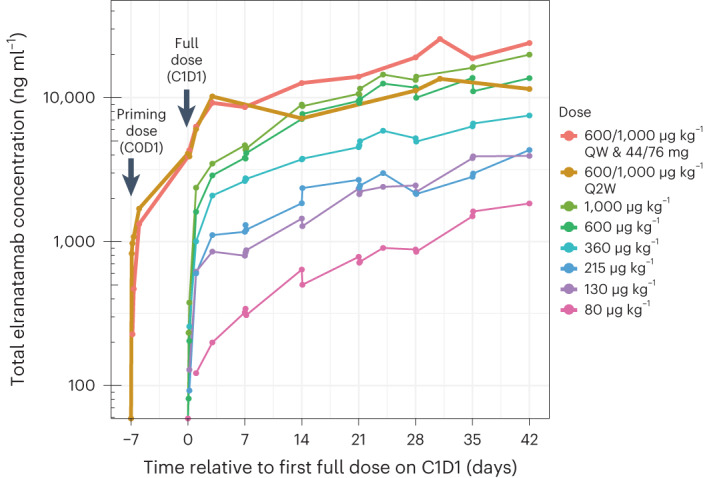
Elranatamab pharmacokinetics. A priming dose was not administered during dose escalation (part 1), whereas a single priming dose of 600 µg kg^−1^ (or equivalent 44-mg fixed dose) was administered 1 week before the RP2D during expansions (part 1.1 and part 2A). C, cycle; D, day.

Elranatamab demonstrated linear pharmacokinetics and low incidence of immunogenicity. Elranatamab concentrations over time (Fig. 2) and pharmacokinetic parameters (Extended Data Table 4) are summarized across all subcutaneous dose levels from 80 µg kg^−1^ to 1,000 µg kg^−1^. Elranatamab showed prolonged absorption, and exposure increased in a dose-proportional manner. A dose of 1,000 µg kg^−1^ every 2 weeks (Q2W) achieved exposure between that observed for 360 μg kg^−1^ once weekly (QW) and 1,000 μg kg^−1^ QW, which were associated with anti-myeloma activity. After subcutaneous dosing, 8.6% (5/58) of patients evaluable for immunogenicity showed treatment-induced anti-drug antibodies (ADAs), including one patient with neutralizing antibodies. All but one patient with ADAs had low titer (close to the minimum required dilution), and no patients who received elranatamab at the RP2D (1,000 μg kg^−1^ or 76 mg QW) developed ADAs.

**Extended Data Fig. 1:**
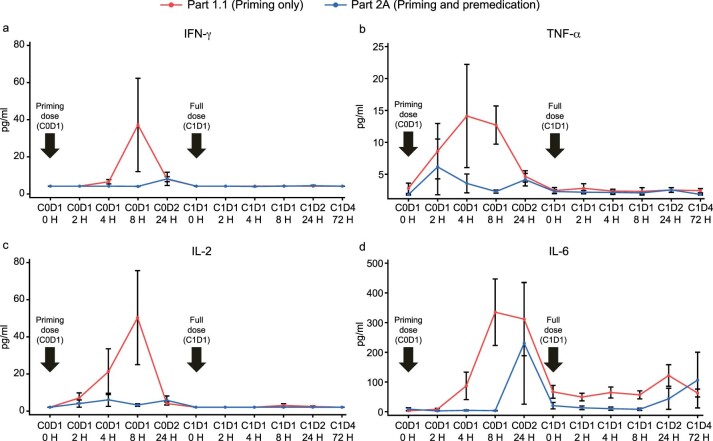
Effect of premedication on cytokine production including (a) IFN-γ, (b) TNF-α, (c) IL-2, and (d) IL-6. The mean is plotted from 20 patients in Part 1.1 and 15 patients in Part 2A. Error bars represent standard error of the mean. C, cycle; D, day; H, hour; IFN-γ, interferon-gamma; IL-2, interleukin 2; IL-6, interleukin 6; TNF-α, tumor necrosis factor-alpha.

Serum cytokines, including those produced by activated T cells, were increased after the first dose of elranatamab. Consistent with mitigation of clinical CRS, elevated serum levels of cytokines, including interferon-gamma, interleukin-2, tumor necrosis factor-alpha and interleukin-6, were observed in part 1.1 after the priming dose but then substantially attenuated at the RP2D (Extended Data Fig. 1a–d). Notably, the addition of dexamethasone-based premedication in part 2A reduced cytokine production associated with the priming dose.

### Efficacy endpoints

**Fig. 3:**
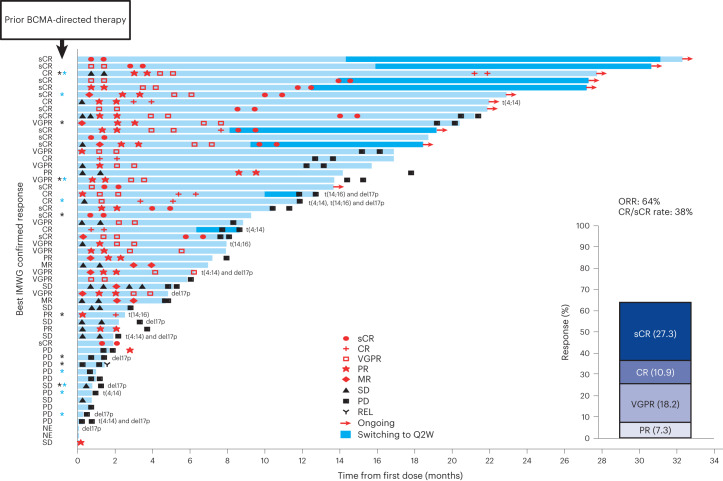
Best overall response and duration of treatment. Swimmer plot depicts disease assessments relevant to first response, confirmation of response, deepening of response and best response. Black asterisk indicates prior anti-BCMA ADC. Blue asterisk indicates prior BCMA-targeted CAR-T. MR, minimal response; NE, not evaluable; PD, progressive disease; REL, relapse; SD, stable disease.

**Fig. 4:**
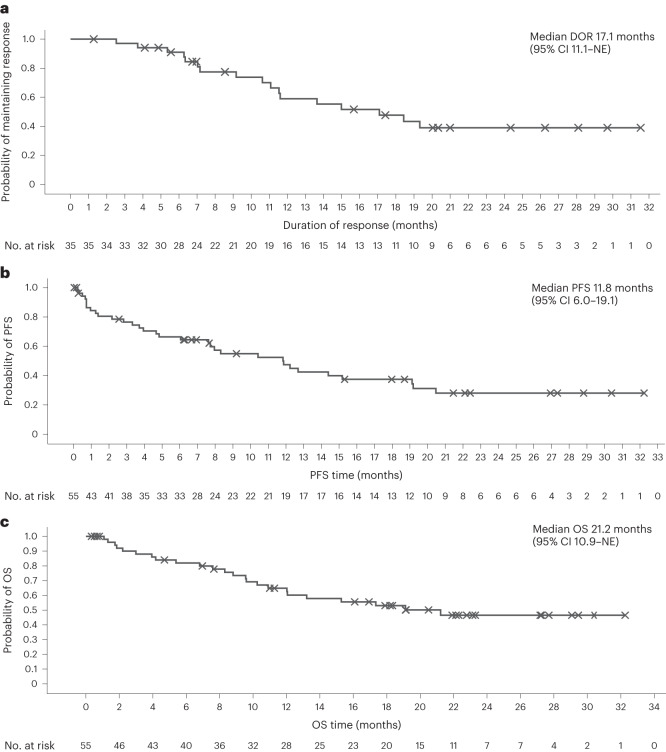
Kaplan–Meier plot. **a**–**c**, Kaplan–Meier plot. for DOR (**a**), PFS (**b**) and OS (**c**). NE, not estimable.

**Extended Data Fig. 2:**
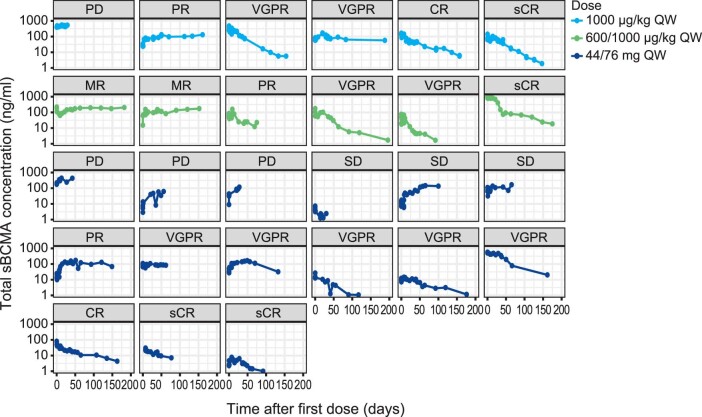
Elranatamab-induced changes in soluble BCMA. BCMA, B-cell maturation antigen; CR, complete response; MR, minimal response; PD, progressive disease; PR, partial response; QW, once weekly; sBCMA, soluble B-cell maturation antigen; sCR, stringent complete response; SD, stable disease; VGPR, very good partial response.

**Extended Data Fig. 3:**
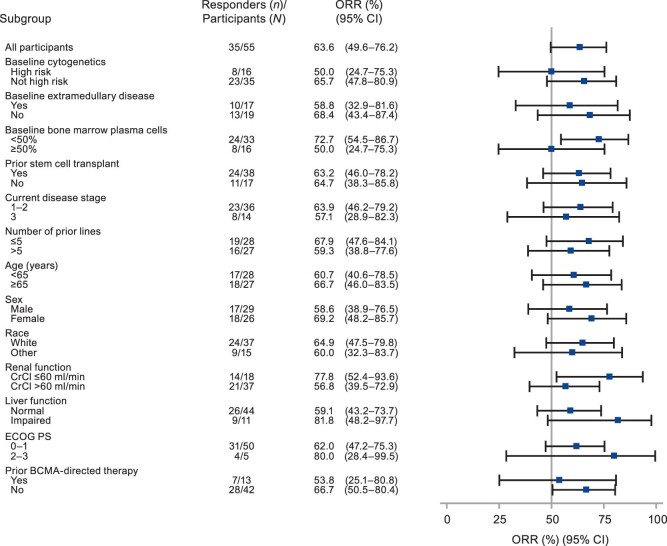
Objective response rate across subgroups. Square denotes ORR and the whiskers indicate 95% CI (Clopper-Pearson). Liver function: normal = AST and total bilirubin ≤ULN; impaired = AST or total bilirubin >ULN (including both AST and total bilirubin >ULN). AST, aspartate aminotransferase; BCMA, B-cell maturation antigen; CI, confidence interval; CrCl, creatinine clearance; ECOG PS, Eastern Cooperative Oncology Group performance status; ORR, objective response rate; ULN, upper limit of normal.

Elranatamab demonstrated anti-myeloma activity and achieved durable clinical and molecular responses. During dose escalation (part 1), the ORR was 50.0% (2/4) at 215 μg kg^−1^, 75.0% (3/4) at 360 μg kg^−1^, 66.7% (4/6) at 600 μg kg^−1^ and 83.3% (5/6) at 1,000 μg kg^−1^. No confirmed responses of PR or better were observed at dose levels less than 215 μg kg^−1^, including the two lowest subcutaneous dose levels (80 μg kg^−1^ and 130 μg kg^−1^) or the intravenous dose levels (all ≤50 μg kg^−1^). The RP2D of 1,000 μg kg^−1^ was established based on an integrated assessment of safety, pharmacokinetics, pharmacodynamics and efficacy. For the 55 patients treated with single-agent elranatamab at efficacious dose levels ≥215 μg kg^−1^, median duration of follow-up was 12.0 months (range, 0.3‒32.3). Overall, the ORR was 63.6% (35/55; 95% confidence interval (CI): 50.4–75.1) with 56.4% (31/55) of patients achieving very good partial response (VGPR) or better and 38.2% (21/55) of patients achieving complete response (CR) or better (Fig. 3). Specifically, 27.3% (15/55) of patients achieved confirmed stringent complete responses (sCRs); 10.9% (6/55) achieved confirmed CR; 18.2% (10/55) achieved confirmed VGPR; and 7.3% (4/55) achieved confirmed PR. Serum levels of soluble BCMA, a potential surrogate for disease burden, decreased over time in responding patients (Extended Data Fig. 2). Among 13 patients with prior BCMA-directed therapy, five patients were refractory, and eight patients had received either ADC (*n* = 5) or CAR-T (*n* = 3) immediately before elranatamab therapy. Notably, 53.8% (7/13) of patients with prior BCMA-directed therapy achieved confirmed responses (two sCR, two CR, two VGPR and one PR), including four patients with prior BCMA-directed therapy immediately before elranatamab therapy. Across all 35 responders, median time to first confirmed response of PR or better was 36.0 d (range, 7‒262), and median duration of response (DOR) was 17.1 months (95% CI: 11.1–not estimable) (Fig. 4a). Of the eight responders who transitioned to less frequent (Q2W) dosing after ≥6 months of QW therapy, 75.0% (6/8) remained on elranatamab therapy and maintained or deepened response with time. All patient subgroups benefited from elranatamab therapy, with a trend toward lower benefit among those with high cytogenetic risk, extramedullary disease, more than 50% plasma cells in bone marrow or prior BCMA-directed therapy (Extended Data Fig. 3). For all 55 patients, median progression-free survival (PFS) was 11.8 months (95% CI: 6.0‒19.1) (Fig. 4b), and median overall survival (OS) was 21.2 months (95% CI: 10.9‒not estimable) (Fig. 4c).

**Fig. 5:**
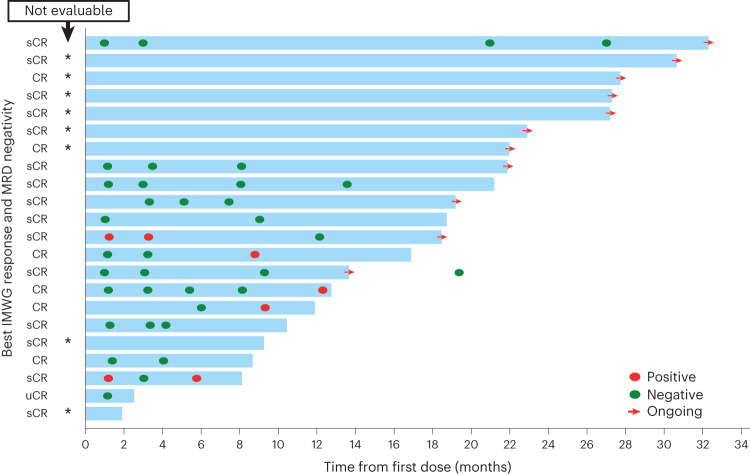
Duration of treatment and molecular response for patients achieving CR or sCR. MRD status was assessed by next-generation sequencing at a sensitivity of 1 × 10^−5^ in accordance with IMWG criteria. Evaluable patients had a dominant VDJ or VJ sequence at baseline and confirmed response of CR or better. Black asterisk indicates ‘not evaluable’. u, unconfirmed.

A total of 13 patients with confirmed CR or better had a dominant variable (V)–diversity (D)–joining (J) or VJ sequence at baseline and were, therefore, minimal residual disease (MRD) evaluable (Fig. 5). Notably, all 13 (100.0%) patients achieved MRD negativity at a sensitivity of 1 × 10^−5^, and nine (69.2%) patients with confirmed CR or better achieved MRD negativity at the 1-month assessment. Molecular responses were durable, and eight (61.5%) patients had sustained MRD negativity beyond 6 months, including two (15.4%) patients with ongoing sCR beyond 2 years.

## Discussion

MagnetisMM-1 is the first-in-human phase 1 study of elranatamab for patients with RRMM. The RP2D of 1,000 μg kg^−1^ (equivalent to fixed dose of 76 mg) is supported by available data on safety, pharmacokinetics, pharmacodynamics and efficacy. Among 55 patients with RRMM who were heavily pretreated and who received single-agent elranatamab subcutaneously at efficacious doses ≥215 μg kg^−1^, the ORR was 63.6%, with 38.2% of patients achieving CR or better. Notably, 90.9% of these patients were triple-class refractory. Elranatamab induced rapid and durable responses, with half of responders maintaining response for more than 17 months. This benefit extended to patients with prior BCMA-directed therapy and across all subgroups, with response rates of ≥50% for those with high cytogenetic risk, extramedullary disease or more than 50% bone marrow plasmacytosis. For patients with confirmed CR or better who were MRD evaluable, all 13 patients achieved MRD negativity, and more than 65% of them achieved MRD negativity at the 1-month assessment. Elranatamab-induced durable molecular responses and sustained MRD negativity beyond 6 months were documented for more than 60% of MRD-evaluable patients. These results are particularly notable in the context of an emerging body of evidence linking MRD status to survival^11^. In addition to inducing durable clinical and molecular responses, patients with RRMM who received elranatamab achieved a median PFS of 11.8 months and a median OS of 21.2 months.

Elranatamab demonstrated a manageable safety profile. DLTs were not observed during dose escalation, and a maximum tolerated dose was not reached. The most common TEAEs regardless of causality included CRS and cytopenias. With premedication and a single priming dose, the overall incidence of CRS was reduced to 66.7% and divided equally between grade 1 and grade 2, with no grade ≥3 events. Similarly, the overall incidence of ICANS was reduced to 13.3% and divided equally between grade 1 and grade 2, with no grade ≥3 events. Notably, subsequent studies with elranatamab implemented a step-up priming dose regimen (12 mg on day 1 and 32 mg on day 4) to further mitigate CRS and ICANS. In the present study, infections were common, and grade 3 and grade 4 events occurred in 21.8% and 5.5% of patients, respectively. These results highlight the importance of patient education, preventive measures^12^, regular monitoring and prompt diagnosis and treatment for infection.

Elranatamab showed predictable pharmacokinetics and low immunogenicity. Exposure increased in a dose-dependent manner and, consistent with maintenance or deepening of response after transition to less frequent (Q2W) dosing after ≥6 months of QW therapy, a dose of 1,000 µg kg^−1^ Q2W achieved exposure in the range associated with anti-myeloma activity. The overall incidence of treatment-induced ADAs was 8.6%, and no patients who received elranatamab at the RP2D QW developed ADAs.

Immunotherapeutic approaches, including both T-cell-engaging bispecific antibodies and CAR-T therapies, have provided important new opportunities for the treatment of patients with MM. Elranatamab represents a readily accessible off-the-shelf therapy with flexibility for biweekly dosing and provides an option for patients requiring immediate treatment or unable to access CAR-T therapies. Results from MagnetisMM-1 support the favorable risk–benefit profile of elranatamab and highlight a particularly compelling combination of attributes even among these emerging immunotherapies^5–8^. In general, CAR-T therapies have response rates in RRMM similar to (ide-cel, 67.1%) or better than (cilta-cel, 83.2%) those achieved by bispecific molecules (teclistamab, 63.0%; elranatamab, 63.6%) but with higher overall incidence (and severity) of toxicities, including CRS (ide-cel, 83.6% and cilta-cel, 94.8%; versus teclistamab, 72.1% and elranatamab, 66.7%). MRD negativity rates among MRD-evaluable patients were high for all four therapies: elranatamab 100.0% (13/13), ide-cel 100.0% (33/33), cilta-cel 94.3% (33/35) and teclistamab 81.5% (44/54). Although there are inherent limitations to comparisons across studies, additional efficacy endpoints for RRMM have been reported, with a median follow-up of approximately 1 year for elranatamab in this first-in-human phase 1 study (MagnetisMM-1) and for two currently approved BCMA-targeted immunotherapies including teclistamab and ide-cel (Extended Data Table 5). Median DOR for elranatamab (17.1 months) was similar to that for teclistamab (18.4 months) and longer than that for ide-cel (10.7 months). Median PFS for elranatamab (11.8 months) was similar to that for teclistamab (11.3 months) and numerically longer than that for ide-cel (8.8 months). Median OS for elranatamab (21.2 months) was similar to that for teclistamab (18.3 months) and ide-cel (19.4 months). These results demonstrate not only the ways in which immunotherapeutic approaches have extended the range of options for patients with RRMM but also the importance of customizing therapy to maximize efficacy and minimize toxicity for individual patients.

The interpretation of the results in this study is limited by its single-arm design and lack of direct comparison with other treatment options as well as by the small sample size in some subgroups. However, elranatamab induced durable clinical and molecular responses with predictable pharmacokinetics and a manageable safety profile for patients with RRMM. These results, along with emerging evidence for both PFS and OS, support the favorable risk–benefit profile of elranatamab at its RP2D (76 mg subcutaneously weekly) for patients with RRMM. Ongoing studies, including the pivotal phase 2 study (MagnetisMM-3), will further investigate elranatamab for patients with RRMM or newly diagnosed MM.

## Methods

### Study design and participants

MagnetisMM-1 is a first-in-human, open-label, multicenter, phase 1 study (NCT03269136) that enrolled patients from November 2017 through April 2021 at 14 investigative centers (11 in the United States and three in Canada). The study included intravenous and subcutaneous dose escalation without priming or premedication (part 1), expansions with priming but no premedication (part 1.1) and expansion with both priming and premedication (part 2A). Eligible patients aged ≥18 years had a diagnosis of MM as defined by IMWG criteria^14^, measurable disease and progression or intolerance to standard therapies, including at least one proteasome inhibitor, immunomodulatory drug and CD38-directed antibody. Prior BCMA-targeted therapy was permitted. Eastern Cooperative Oncology Group performance status (ECOG PS) of 0–1 or 2 (if due to underlying MM) as well as adequate hepatic (total bilirubin ≤2.0 mg dl^−1^ with exception for Gilbert syndrome; alkaline phosphatase and aspartate/alanine aminotransferases ≤2.5 times the upper limit of normal with exceptions for bone or liver involvement by tumor, respectively), renal (creatinine clearance ≥30 ml min^−1^) and hematopoietic (absolute neutrophil count ≥1,000 mm^3^, platelet count ≥25,000 mm^3^ and hemoglobin ≥8.0 g dl^−1^) function were required.

This study was conducted in accordance with the Declaration of Helsinki and the International Conference on Harmonization guidelines for Good Clinical Practice. All patients provided written informed consent. The study protocol and relevant documents were approved by an independent institutional review board or ethics committee at each investigative center. Patient safety was monitored jointly by investigators and a safety assessment committee established by the sponsor.

### Procedures

Patients received elranatamab monotherapy intravenously (dose levels 0.1, 0.3, 1, 3, 10, 30 or 50 μg kg^−1^) or subcutaneously (dose levels 80, 130, 215, 360, 600 or 1,000 μg kg^−1^) either QW or Q2W until disease progression, withdrawal of consent, death or discontinuation. To mitigate CRS, a single priming dose (600 μg kg^−1^ or equivalent 44-mg fixed dose) was administered to patients who received elranatamab at the RP2D (1,000 μg kg^−1^ or equivalent 76-mg fixed dose) in part 1.1 (*n* = 20) and part 2A (*n* = 15). In addition, patients enrolled in part 2A received premedication (dexamethasone 20 mg or equivalent, antihistamine and antipyretic) before the priming dose and the first full treatment dose. Dose modifications were permitted for management of adverse events. Patients with disease stability for ≥2 months were permitted to transition to elranatamab Q2W after 6 months of QW therapy.

### Outcomes

For dose escalation (part 1), the primary endpoint was the number of DLTs. The primary efficacy endpoints were ORR and DOR for patients treated at efficacious doses, with response assessed according to IMWG criteria^15^. Secondary endpoints included adverse events, laboratory abnormalities, ORR, time to response, CR rate, DOR, PFS, OS, rate of MRD negativity, pharmacokinetic parameters, immunogenicity and levels of serum cytokines. Additional planned secondary endpoints not reported in this manuscript included above-described endpoints in patients treated with elranatamab in combination with immunomodulatory agents. Exploratory endpoints included levels of soluble BCMA and characterization of immune cells in whole blood and bone marrow by flow cytometry analysis.

The DLT observation period was through the end of the first treatment cycle for each patient in part 1. TEAEs were graded according to National Cancer Institute Common Terminology Criteria for Adverse Events (NCI CTCAE) version 4.03. Both CRS and ICANS were defined and graded according to American Society for Transplantation and Cellular Therapy consensus criteria^13^. Tumor response and disease progression were assessed according to IMWG response criteria^15^, and ORR was calculated based on confirmed responses reported by investigators. MRD at a sensitivity of 1 × 10^−5^ was centrally assessed by next-generation sequencing (clonoSEQ, Adaptive Biotechnologies) according to IMWG response criteria^15^. Pharmacokinetics, cytokines, lymphocyte subsets and serum levels of soluble BCMA were analyzed over time.

### Statistical analysis

Safety and efficacy were evaluated in all patients enrolled who received at least one dose of elranatamab. Elranatamab dose escalation was guided using a Bayesian method with modified Toxicity Probability Interval design^16^. Maximum tolerated dose was defined as the dose with approximately 25% probability of DLT and considers equivalent doses that yield a probability of DLT in the (equivalence) interval between 20% and 30%. Due to the dynamic nature of the Bayesian allocation procedure, the sample size of the modified toxicity probability interval could not be determined in advance. No formal hypothesis testing was performed for efficacy endpoints.

Summary statistics for categorical variables were reported with mean (s.d.) or median (95% CI or range) unless otherwise specified. Time-to-event endpoints were analyzed using the Kaplan–Meier method^17^. CIs for medians were calculated according to the Clopper–Pearson method^18^, and CIs for Kaplan–Meier estimates were derived using the log(−log) method^19^. SAS version 9.4 software was used for statistical analysis. This ongoing study is registered with ClinicalTrials.gov (NCT03269136).

### Reporting summary

Further information on research design is available in the Nature Portfolio Reporting Summary linked to this article.

## Online content

Any methods, additional references, Nature Portfolio reporting summaries, source data, extended data, supplementary information, acknowledgements, peer review information; details of author contributions and competing interests; and statements of data and code availability are available at 10.1038/s41591-023-02589-w.

### Supplementary information

Reporting Summary

## Figures and Tables

**Extended Data Table 1:**
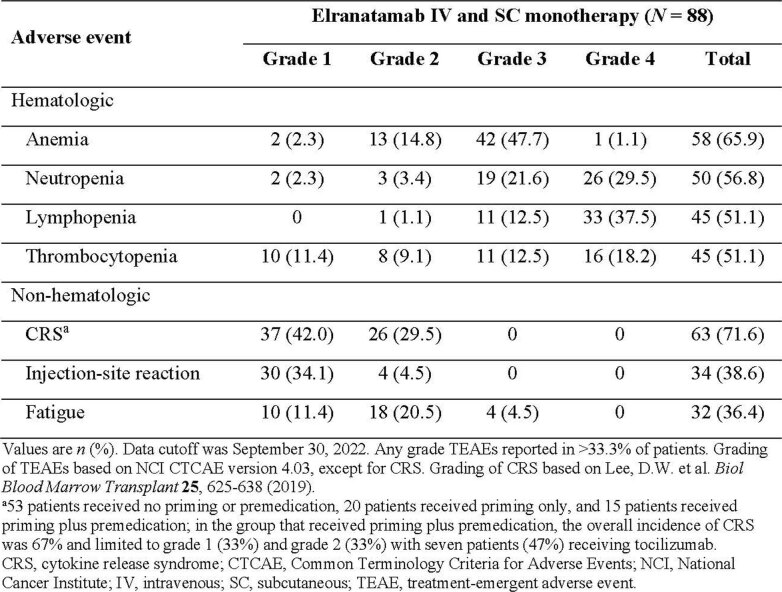
TEAEs (all causality)

**Extended Data Table 2:**
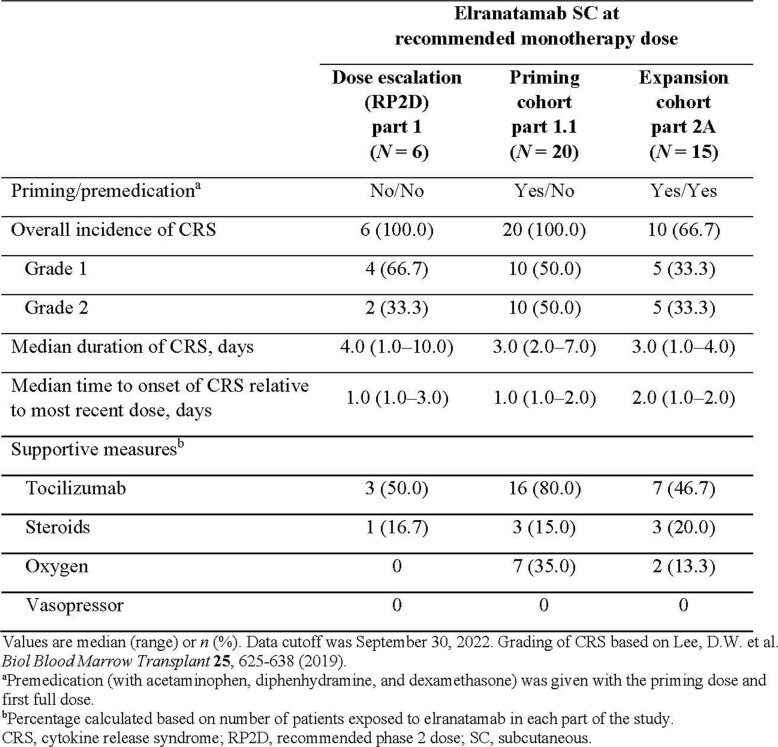
Effect of priming and premedication on CRS

**Extended Data Table 3:**
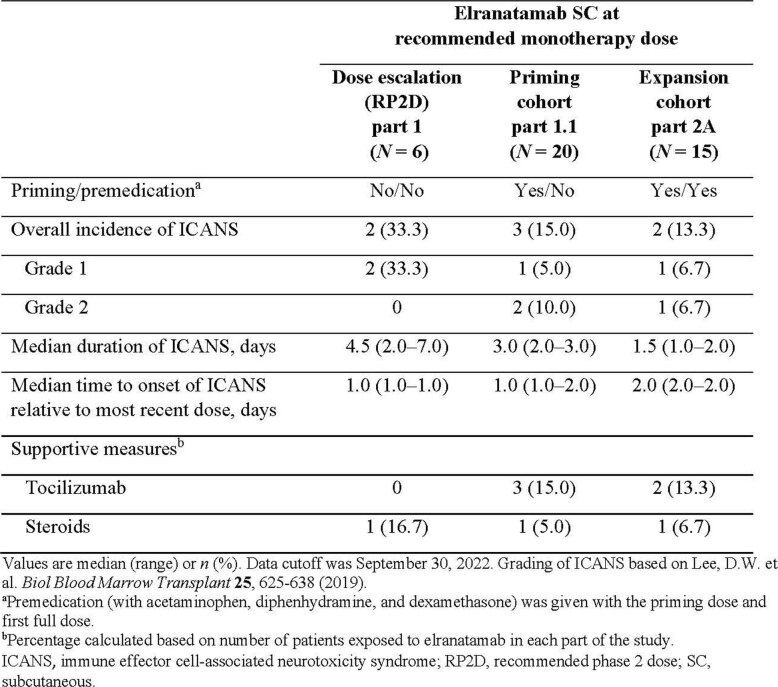
Effect of priming and premedication on ICANS

**Extended Data Table 4:**
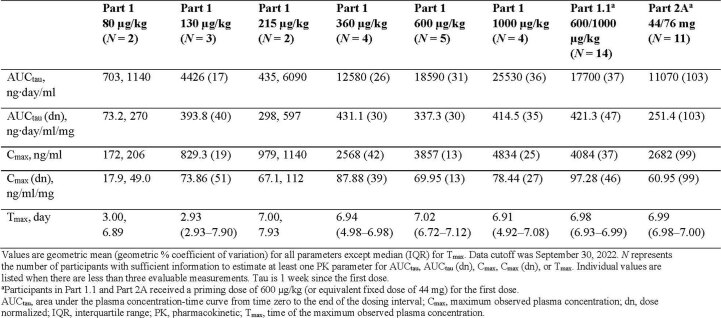
Elranatamab serum pharmacokinetic (total) parameters 1 week after a single subcutaneous dose administration

**Extended Data Table 5:**
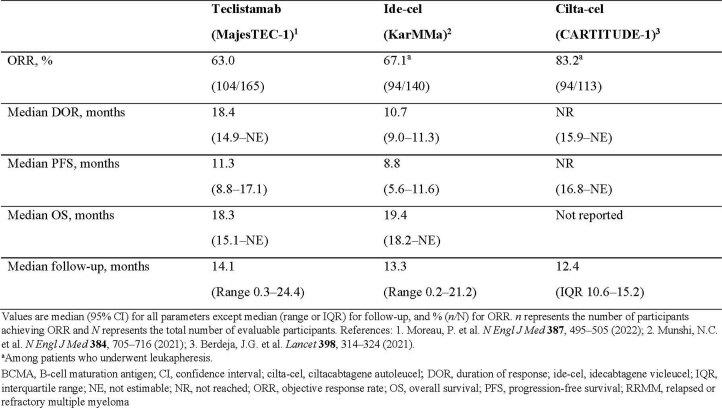
Efficacy endpoints reported in RRMM for currently approved BCMA-targeted immunotherapies

## Data Availability

Upon reasonable request, and subject to review, Pfizer will provide the data that support the findings of this study. Subject to certain criteria, conditions and exceptions, Pfizer may also provide access to the related individual de-identified participant data. See https://www.pfizer.com/science/clinical-trials/trial-data-and-results for more information. The protocol and statistical analysis plan for MagnetisMM-1 have been uploaded to ClinicalTrials.gov.
