# Supplementary material for: Elranatamab in relapsed or refractory multiple myeloma: the MagnetisMM-1 phase 1 trial
Source: Nat Med. 2023 Oct 2;29(10):2570–6. doi: 10.1038/s41591-023-02589-w (PMC10579053; doi:10.1038/s41591-023-02589-w)
Supplement: Supplementary file 1 — Reporting Summary [file 41591_2023_2589_MOESM1_ESM.pdf]

nature portfolio

Corresponding author(s): Nizar J. Bahlis

Last updated by author(s): Sep 7, 2023

Reporting Summary

Nature Portfolio wishes to improve the reproducibility of the work that we publish. This form provides structure for consistency and transparency in reporting. For further information on Nature Portfolio policies, see our Editorial Policies and the Editorial Policy Checklist.

Statistics

For all statistical analyses, confirm that the following items are present in the figure legend, table legend, main text, or Methods section.

n/a	Confirmed	
☐	☒	The exact sample size (n) for each experimental group/condition, given as a discrete number and unit of measurement
☒	☐	A statement on whether measurements were taken from distinct samples or whether the same sample was measured repeatedly
☐	☒	The statistical test(s) used AND whether they are one- or two-sided<br>*Only common tests should be described solely by name; describe more complex techniques in the Methods section.*
☒	☐	A description of all covariates tested
☒	☐	A description of any assumptions or corrections, such as tests of normality and adjustment for multiple comparisons
☐	☒	A full description of the statistical parameters including central tendency (e.g. means) or other basic estimates (e.g. regression coefficient) AND variation (e.g. standard deviation) or associated estimates of uncertainty (e.g. confidence intervals)
☒	☐	For null hypothesis testing, the test statistic (e.g. F, t, r) with confidence intervals, effect sizes, degrees of freedom and P value noted<br>*Give P values as exact values whenever suitable.*
☒	☐	For Bayesian analysis, information on the choice of priors and Markov chain Monte Carlo settings
☒	☐	For hierarchical and complex designs, identification of the appropriate level for tests and full reporting of outcomes
☒	☐	Estimates of effect sizes (e.g. Cohen's d, Pearson's r), indicating how they were calculated

Our web collection on statistics for biologists contains articles on many of the points above.

Software and code

Policy information about availability of computer code

Data collection	Oracle Clinical Remote Data Capture (OCRDC) 5.4.0
Data analysis	SAS version 9.4.

For manuscripts utilizing custom algorithms or software that are central to the research but not yet described in published literature, software must be made available to editors and reviewers. We strongly encourage code deposition in a community repository (e.g. GitHub). See the Nature Portfolio guidelines for submitting code & software for further information.

Data

Policy information about availability of data

All manuscripts must include a data availability statement. This statement should provide the following information, where applicable:

- Accession codes, unique identifiers, or web links for publicly available datasets
- A description of any restrictions on data availability
- For clinical datasets or third party data, please ensure that the statement adheres to our policy

The URL in the current statement includes the most up-to-date information, including time frame, restrictions contact details, etc., with respect to data request. The statement is: "Upon request, and subject to review, Pfizer will provide the data that support the findings of this study. Subject to certain criteria, conditions, and exceptions, Pfizer may also provide access to the related individual de-identified participant data. See https://www.pfizer.com/science/clinical-trials/trial-data-and-results for more information. The protocol and statistical analysis plan for MagnetisMM-1 have been uploaded to clinicaltrial.gov."

Research involving human participants, their data, or biological material

Policy information about studies with human participants or human data. See also policy information about sex, gender (identity/presentation), and sexual orientation and race, ethnicity and racism.

Reporting on sex and gender	Information on sex was obtained for all study participants and is detailed in Table 1. 26 (47.3) participants were female, and 29 (52.7%) participants were male. No information about gender was collected. No analysis was performed based on sex since this was not the focus of this study.
Reporting on race, ethnicity, or other socially relevant groupings	Information on race was obtained for all study participants and is detailed in Table 1. No analysis was performed based on race.
Population characteristics	No covariate analysis was conducted. The baseline characteristics of study participants are detailed in Table 1.
Recruitment	Patients were recruited by participating investigators. Investigators obtained written informed consent from each patient before any study-specific activity was performed. A total of 14 investigative centers (11 in the USA, 3 in Canada) enrolled patients from November 2017 through April 2021. Due to the geographical distribution of the study centers, participants may not represent the global general population. No other bias emerging from recruitment is expected.
Ethics oversight	This study was conducted in accordance with the Declaration of Helsinki and the International Conference on Harmonization guidelines for Good Clinical Practice. Informed consent documents and patient recruitment materials were compliant with International Conference on Harmonization Good Clinical Practice, local regulatory requirements, and legal requirements, including applicable privacy laws. The study protocol and relevant documents were approved by an independent institutional review board or ethics committee at each investigative center. Patient safety was monitored jointly by investigators and a safety assessment committee established by the sponsor.

Note that full information on the approval of the study protocol must also be provided in the manuscript.

Field-specific reporting

Please select the one below that is the best fit for your research. If you are not sure, read the appropriate sections before making your selection.

☒ Life sciences ☐ Behavioural & social sciences ☐ Ecological, evolutionary & environmental sciences

For a reference copy of the document with all sections, see nature.com/documents/nr-reporting-summary-flat.pdf

Life sciences study design

All studies must disclose on these points even when the disclosure is negative.

Sample size	This phase 1 trial utilized modified toxicity probability interval procedure for patient allocation and hence the sample size was not pre-determined. This is typical for phase 1 dose-finding clinical trials.
Data exclusions	Data for patients who received intravenous therapy and subcutaneous therapy at sub-efficacious dose levels not associated with International Myeloma Working Group confirmed responses of partial response or better were excluded from the analysis presented in the article.
Replication	Replication is not applicable for human clinical trial as each patient is individual and inherently different.
Randomization	This was a non-randomized phase 1 study. Data collection was prospective without controling for covariates.
Blinding	This was an open-label study. Participants were not randomized.

Reporting for specific materials, systems and methods

We require information from authors about some types of materials, experimental systems and methods used in many studies. Here, indicate whether each material, system or method listed is relevant to your study. If you are not sure if a list item applies to your research, read the appropriate section before selecting a response.

Materials & experimental systems

n/a	Involved in the study
☒	☐ Antibodies
☒	☐ Eukaryotic cell lines
☒	☐ Palaeontology and archaeology
☒	☐ Animals and other organisms
☐	☒ Clinical data
☒	☐ Dual use research of concern
☒	☐ Plants

Methods

n/a	Involved in the study
☒	☐ ChIP-seq
☒	☐ Flow cytometry
☒	☐ MRI-based neuroimaging

Clinical data

Policy information about clinical studies

All manuscripts should comply with the ICMJE guidelines for publication of clinical research and a completed CONSORT checklist must be included with all submissions.

Clinical trial registration

NCT03269136

Study protocol

Information can be accessed at : https://www.clinicaltrials.gov/ct2/show/NCT03269136 or by reviewing the study protocol in the Supporting Information.

Data collection

Patients were enrolled from 14 investigative centers from November 2017 through April 2021; the data cutoff was September 30, 2022.

Outcomes

As defined in the protocol (accessible via URL above), the primary safety endpoint was the number of dose-limiting toxicities; the primary efficacy endpoints were objective response rate and duration of response for patients treated at efficacious doses, with response assessed according to International Myeloma Working Group (IMWG) criteria. Secondary endpoints included adverse events, laboratory abnormalities, objective response rate, time to response, complete response rate, duration of response, progression-free survival, overall survival, rate of minimal residual disease negativity, pharmacokinetic parameters, immunogenicity, and levels of serum cytokines.

Primary and secondary endpoints were predefined in the protocol before the trial began enrollment. They were assessed as per description in the methods section, including the following: "The dose-limiting toxicity observation period was through the end of the first treatment cycle for each patient in Part 1. Treatment-emergent adverse events were graded according to National Cancer Institute Common Terminology Criteria for Adverse Events version 4.03. Both cytokine release syndrome and immune effector cell-associated neurotoxicity syndrome were defined and graded according to American Society for Transplantation and Cellular Therapy consensus criteria. Tumor response and disease progression were assessed according to IMWG response criteria, and objective response rate was calculated based on confirmed responses reported by investigators. Minimal residual disease at a sensitivity of 1×10–5 was centrally assessed by next-generation sequencing (clonoSEQ®, Adaptive Biotechnologies, Seattle, WA, USA) according to IMWG response criteria. Pharmacokinetics, cytokines, lymphocyte subsets, and serum levels of soluble B-cell maturation antigen were analyzed over time."
